# Hepatitis Viruses in Heamodialysis Patients: An Added Insult to Injury?

**DOI:** 10.1155/2013/860514

**Published:** 2013-03-06

**Authors:** Kranthi Kosaraju, Sameer Singh Faujdar, Aashima Singh, Ravindra Prabhu

**Affiliations:** ^1^Department of Microbiology, Kasturba Medical College and Hospital, Manipal University, Madhav Nagar, Manipal 576104, Karnataka, India; ^2^Department of Nephrology, Kasturba Medical College and Hospital, Manipal University, Madhav Nagar, Manipal 576104, Karnataka, India

## Abstract

Hepatitis B (HBV) and hepatitis C (HCV) viruses are the most important causes of chronic liver disease in patients with end stage renal disease on hemodialysis. The prevalence of hepatitis infection among hemodialysis patients is high and varies between countries and between dialysis units within a single country. This case-control study was undertaken to estimate the occurrence of HBV and HCV infections in patients undergoing hemodialysis in our tertiary care center. All patients receving hemodialysis at our centre with HCV or HBV infection were included in the study. The total number of patients admitted for hemodialysis during the study period was 1710. Among these, 26 patients were positive for HBV, 19 were positive for HCV, and 2 were positive for both HCV and HBV. Mean age of the infected cases in our study was 48.63 years. Mean duration of dialysis for infected cases was 4.8 years while that of the noninfected controls was 3.18 years. The mean dialysis interval was twice a week. Interventions to reduce the occurrence of these infections are of utmost need to reduce the risk of long-term complications among hemodialysis patients.

## 1. Introduction

Hepatitis B virus (HBV) and hepatitis C virus (HCV) infections cause morbidity and mortality in haemodialysis patients. Prolonged vascular exposure and multiple blood transfusions increase the risk of acquiring these blood-borne infections in these patients. Contaminated devices, equipments, and supplies, environmental surfaces, and attending personnel may also play a crucial role in the nosocomial transmission of these infections. Infections with hepatitis viruses in haemodialysis patients are further promoted by the significant immune status dysfunction developing due to irreversible renal compromise [1–3].

Furthermore, hepatitis viral infections in haemodialysis patients cause liver disease in renal failure patients undergoing replacement therapy. They also pose a significant problem in the management of these cases as patients with renal failure cannot clear the viruses effectively. Patients with coinfections with these viruses develop severe clinical presentations and resistance to interferon treatment [3].

There are very limited data available on the occurrence of such infections in haemodialysis patients from this part of the country. The present study aimed to investigate the occurrence of HBV and HCV infections in haemodialysis patients and the risk factors associated with such infections.

## 2. Materials and Methods

This study was conducted as a retrospective case-control study involving the haemodialysis patients at a dialysis centre of a tertiary care hospital. All the patients who underwent haemodialysis from January 2004 to June 2012 were included in the study. 

Patients receiving haemodialysis were considered as a “case” for the study if their serum tested positive for either HBV or HCV. In contrast, the patients receiving haemodialysis were considered as a “control” if their serum tested negative for all the three viruses. For every case, one age- and gender-matched control receiving haemodialysis was selected.

Patients' medical records were reviewed to obtain details like age, gender, clinical diagnosis, and duration and frequency of dialysis, history of blood transfusions, and past surgeries and these details were recorded in a preformed questionnaire for all cases and controls. Further, the results of serological tests (HBsAg, Anti-HCV antibodies), renal function tests (serum urea and creatinine), and liver function tests were recorded for both groups. The results of biochemical parameters were correlated with serological findings. 

Duration of dialysis, frequency of dialysis, and the results of biochemical parameters were compared for infected cases and non-infected controls and were analyzed statistically using nonparametric tests and chi-square tests.

## 3. Results

The present retrospective study was conducted for a period of 102 months, that is, from January 2004 to June 2012. A total of 180 patients were diagnosed to have chronic kidney disease and were on maintenance haemodialysis during the study period. Also, our dialysis unit caters to approximately 15 in-patients per month referred from other departments or hospitals for haemodialysis. Therefore, the total number of patients who received haemodialysis at our centre during the study period was 1710.

Among these patients, 45/1710 cases (2.63%) were found to be infected with either HBV or HCV and were included as cases for our study. 

Out of 45 cases studied, 26/1710 (1.52%) tested positive for HBsAg and 19/1710 (1.11%) tested positive for Anti-HCV antibodies. Out of 26 cases infected with HBV, 22 (84.6%) were males and 4 (15.4%) were females. Among HCV positive cases, 18 (94.7%) were males and 1 (5.2%) was female. Among the cases, dual infection with HBV and HCV was seen in two patients, 1 male and 1 female.

Mean age of the infected cases in our study was 48.63 years. HBV infection was seen most commonly in the age group of 50–60 yrs, whereas HCV was commonest in the age group of 30–40 years. 

Mean duration of dialysis for infected cases was 4.8 years while that of the non-infected controls was 3.18 years. There was a statistically significant (*P* = 0.008) difference between the cases and controls with respect to duration of dialysis (Mann-Whitney *U* test since mean was less than 2 standard deviations, non-parametric tests were used). When comparing frequency of dialysis between cases and controls, as it was a categorical variable, chi-square test was applied and there was no statistically significant (*P*  value = 0.228) correlation of the frequency of dialysis between cases and controls.

With respect to liver function tests, a significant elevation of AST (aspartate aminotransferase) and ALT (alanine transaminase) values among infected cases (*P*  value = 0.001) was observed in this study. 

**Figure 1:**
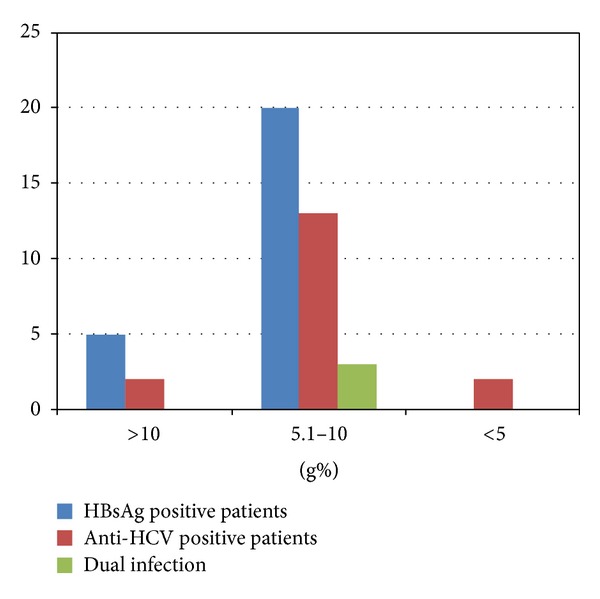
Hemoglobin values in cases infected with HBV and HCV.

**Figure 2:**
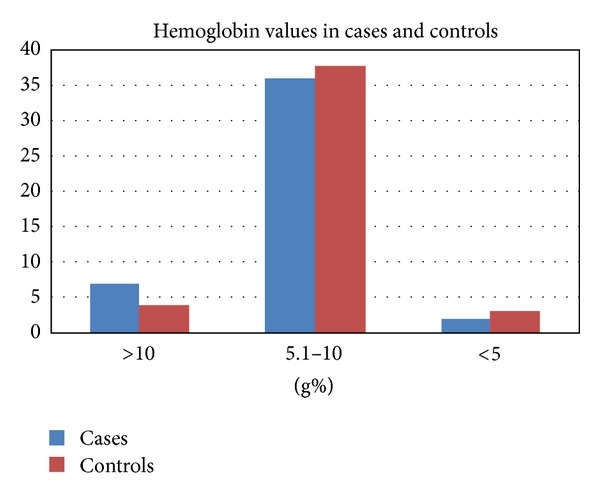
Comparison of hemoglobin values among cases and controls (*n* = 45).

Most of the patients (80%) on haemodialysis in our study were anaemic with haemoglobin concentration <10 g% as shown in Figure 1. The comparison of haemoglobin values among cases and controls is depicted in Figure 2. With respect to renal function tests in cases and controls, mean value of blood urea for cases was 99.85 and for controls was 103.1, and mean value of creatinine was 22.3 for cases and 8.65 for controls, respectively. No statistically significant difference was observed between both groups with respect to serum levels of urea (*P*  value = 0.6), creatinine (*P*  value = 0.228), and haemoglobin (*P*  value = 0.6). 

**Figure 3:**
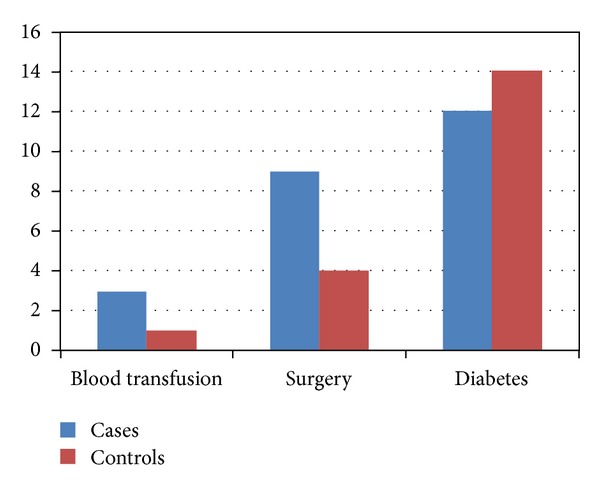
Risk factors among cases and controls.

Risk factors for acquiring HBV and HCV infections were studied for both groups. Among the infected cases, multiple blood transfusions were seen only in 3 cases (2 positive for HBsAg and 1 for HCV). Among the three, one case with positive Anti-HCV underwent multiple blood transfusions and in the remaining two cases, blood transfusion was received only twice. The history of undergoing surgeries was obtained in nine cases. Among the 9 cases, HBV infection was seen in 4, HCV in 4, and dual infection was seen in one case. Twelve cases were diabetic, with HBV infection in 6, HCV in 5 cases, and dual infection in 1 case (Figure 3).

With respect to similar risk factors in the control group, 14 control patients had diabetes and 4 of them had history of undergoing surgery in the past. Only 1 control patient had history of undergoing blood transfusion (Figure 3).

## 4. Discussion

Patients diagnosed with chronic renal failure (CRF) on maintenance haemodialysis pose a higher risk for acquiring HBV or HCV infections due to frequent use of blood and blood products and multiple invasive procedures performed in these patients [1]. The literature review points to the fact that viral hepatitis is a serious threat for haemodialysis patients as 1.9% of all deaths among this population were related to the consequence of viral hepatitis [4].

The results from our study demonstrate that the occurrence of HBV and HCV infections in haemodialysis patients is 1.52% and 1.11%, respectively, which is lower than the rates reported from different studies all over the world and India [5–8]. An Indian study has reported the occurrence of HBV in haemodialysis to vary from 3.4% to 42%, much higher than that seen in our study [9]. The lower rates of infection in our study might be due to decreased transfusion requirements owing to the availability of erythropoietin and better screening of blood and blood products for blood-borne infections. Introduction of vaccination for HBV, isolation of hepatitis B virus (HBV) positive patients, and regular surveillance for HBV infection at our centre could also have contributed for the lesser rates of infection. 

Occurrence of HCV infection was much less than HBV in our study which was in accordance with a study conducted in Spain which reported lesser prevalence rates for HCV infection in haemodialysis (HD) patients [10]. Another study showed a significant decline of hepatitis C infection among end-stage renal disease patients in Central Brazil, highlighting the importance of public health strategies such as screening for anti-HCV in blood banks and infection control measures for control and prevention of hepatitis C in the haemodialysis environment [11]. 

Since both of these viruses share a common mode of transmission, we looked for the occurrence of coinfections among the cases studied. Among the cases, dual infection with HBV and HCV was seen in two patients, 1 male and 1 female (2/45 = 4.4%). Study from the same centre on the occurrence of coinfections in the general population reported lower rates (1.68%) of dual infections [12]. Studies from other centres in India have reported a varying prevalence of coinfections with HBV and HIV (9–40%) and for HCV and HIV (2–8%) [13]. 

The higher occurrence in our study may be due to the study population being restricted to haemodialysis patients and also due to the lesser sample size in the current study. Another factor contributing to the higher rates is the enhanced risk of coinfections among chronic renal failure patients on haemodialysis due to multiple transfusions and invasive procedure performed in these patients [14]. However, dual infection with HBV and HCV in our study which was 4.4%, similar to the study conducted in Hyderabad [3]. 

The present study highlights the duration of dialysis as an important risk factor for infection among haemodialysis patients. This observation was in agreement with previous reports in Palestine, Moldavia, and other studies from different regions of the world [15–17]. Duration of dialysis is an important risk factor for acquiring infections as it is related to nosocomial transmission and dissemination of the infections in the dialysis units [1].

On comparison of AST and ALT values among cases and controls, it was found that cases had higher mean value. This can be explained on the basis of destruction of hepatocytes caused by the immune reaction of body responding to the hepatitis viruses leading to excessive release of the aminotransferases. These findings were in accordance with the studies conducted in Karachi and Jenin district and Gaza strip in Palestine [1, 18].

In our study, when compared with the control group, 67% of the patients on haemodialysis with HBV/HCV were found to have higher hemoglobin levels. This interesting finding was supported by few studies and was attributed to the increase in RBC counts after infection with hepatitis viruses and also to increased erythropoietin production after hepatic stimulation by chronic infection with hepatitis virus. However, the exact cause for the same is not yet established [19–22].

## 5. Conclusion

A study from our centre brings to light that HBV and HCV infections, though less common, continue to remain as an important causes of infection in haemodialysis patients. The risk of exposure increases with the number of dialysis sessions and is maximum in patients on maintenance haemodialysis. Interventions to reduce the occurrence of these infections are of utmost need to reduce the risk of long-term complications.
